# Cognitive processing therapy for posttraumatic stress disorder in first responders and veterans: Flexing the approach with explicit case formulation

**DOI:** 10.1002/jts.70005

**Published:** 2025-09-14

**Authors:** Reginald D. V. Nixon, David Forbes, Tara E. Galovski

**Affiliations:** ^1^ College of Education, Psychology and Social Work, Flinders Institute for Mental Health and Wellbeing Flinders University Adelaide Australia; ^2^ Department of Psychiatry, Phoenix Australia Centre for Posttraumatic Mental Health University of Melbourne Melbourne Australia; ^3^ National Center for PTSD, VA Boston Healthcare System Boston Massachusetts USA; ^4^ Boston University Chobanian & Avedisian School of Medicine Boston Massachusetts USA

## Abstract

There is a need to improve psychological interventions for first responders and veterans with posttraumatic stress disorder (PTSD). We conducted an open trial integrating explicit case formulation (CF) within cognitive processing therapy (CPT) in a sample primarily composed of first responders (*N* = 29). Participants attended weekly CPT sessions with explicit CF, where CF guided deviations (if required) from standard CPT delivery (CPT‐CF). PTSD diagnosis and self‐reported PTSD symptoms, depressive symptoms, and quality of life utility scores were key variables assessed at pretreatment, posttreatment, and 3‐month follow‐up for all participants. Of the 28 participants who started therapy, 23 completed treatment. Intent‐to‐treat analyses indicated significant reductions and sizeable effects at posttreatment for clinician‐rated and self‐reported PTSD outcomes, *g* = 2.48–2.50, and self‐reported depressive symptoms, *g* = 1.37, and quality of life, *g* = 0.99. Effects for secondary variables ranged from small (alcohol misuse: *g* = 0.32) to large (sleep, *g* = 0.71; anger, *g* = 0.74; unhelpful trauma beliefs: *g* = 1.11). Clinical gains were maintained at 3‐month follow‐up. Among the 23 participants available at follow‐up, 82.6% (*n* = 19) met good end‐state functioning for PTSD, and none met the criteria for PTSD. Seven participants had moderate‐to‐major deviations from CPT during treatment but largely demonstrated similar outcomes to those who did not. The study replicates prior CPT‐CF work among civilians, finding it to be acceptable to participants and not diluting positive outcomes of standard CPT. Future research requires randomized trials and an expansion of this approach with other trauma populations.

Posttraumatic stress disorder (PTSD) is a debilitating and multifaceted condition, often accompanied by a variety of co‐occurring or comorbid physical and psychiatric problems. PTSD has far‐reaching consequences that can negatively impact individuals’ daily functioning, quality of life, social relationships, work‐related productivity, mortality, and morbidity (Bonfils et al., 2022; Monson et al., 2017; Pacella et al., 2013). In recent years, there has been an increased research focus on improving the uptake, delivery, and effectiveness of first‐line PTSD treatments, and efforts in this area continue.

There are numerous effective evidence‐based psychotherapies (EBPs) for the treatment of PTSD (Bradley et al., 2005; Cusack et al., 2016); however, response rates vary, with it recently estimated that 39.2% of individuals continue to suffer from PTSD following treatment (Semmlinger et al., 2024). Moreover, disengagement rates from trauma‐focused therapies remain a concern, with a recent meta‐analysis finding an average disengagement rate of 20.9% (95% CI [17.2, 24.9]; Varker et al., 2021) across RCTs, with this figure reaching as high as 34% for cognitive processing therapy (CPT), one of the most effective treatment options for PTSD (Cusack et al., 2016; Resick, LoSavio, et al., 2024). These factors are compounded by the fact that, generally, clinicians are poor at predicting and detecting who is at risk of dropout or disengagement (Hannan et al., 2005; Hatfield et al., 2010). In addition, clinicians often do not use evidence‐based protocols due to the perception that this approach is inflexible or does not address “real‐world” clients and their comorbidities, despite substantial evidence to the contrary (Becker et al., 2004; Cook et al., 2014; LoSavio et al., 2024; Finch et al., 2020).

PTSD is a difficult clinical presentation to treat, with a range of challenges to optimal therapy outcomes (COTO) that include comorbidities, motivation, and clients’ internal resources (emotional, coping capacity) and external resources (e.g., insecure housing), to name a few (Galovski et al., 2020). Evidence also suggests that certain subpopulations might be at higher risk for PTSD, such as individuals in occupations characterized by chronic exposure to traumatic events. Veterans, military personnel, and first responders experience PTSD at a higher rate than the general public and face unique barriers to seeking and engaging in treatment, including perceived stigma, confidentiality concerns, and workplace norms of self‐reliance and stoicism (O'Dare et al., 2024; Randles & Finnegan, 2022). Fortunately, these populations have been shown to respond to EBPs for PTSD (Alshahrani et al., 2022; Bryant et al., 2019; Peterson et al., 2021). However, research with veteran and military personnel suggests that the benefits seen are much more modest compared to civilian populations (Lehavot et al., 2018; Straud et al., 2019), and in a representative survey of first responders, over 20% of participants with probable PTSD and/or high levels of psychological distress reported delaying seeking help by more than a year (Beyond Blue, 2018). Moreover, once in treatment, numerous individual‐level COTOs are present in these populations, such as high levels of avoidance, problematic alcohol use, suicidal ideation, and impairment in social functioning (Díaz‐Tamayo et al., 2022; Pietrzak et al., 2011; Stanley et al., 2016; Tomaka et al., 2017). These factors can present clinical challenges in the treatment of PTSD, adding to the already complex landscape of the disorder.

Considering these clinician‐ and client‐level challenges, recent research has sought to increase the flexibility of existing first‐line PTSD treatments with the goal of addressing client complexities and ultimately increasing treatment effectiveness. CPT is one therapy that has been subject to this type of investigation, including efforts to augment or expand the intervention with additional techniques to address comorbidities (e.g., depression, sleep problems; Angelakis et al., 2020; Galovski et al., 2016; Walter et al., 2023) or enhance it, for example, by adding sessions to extend the length of treatment (historically studied as a 12‐session protocol) with the option of addressing emergency, non‐PTSD–related stressors as needed (Galovski et al., 2012). This type of research is helping show how, with judicious adjustment, CPT can be used quite flexibly without compromising its efficacy (Galovski, Nixon, & Kehle‐Forbes, 2024).

Given the complex nature of PTSD presentations—there are over 600,000 symptom combinations that meet the criteria for PTSD (Galatzer‐Levy & Bryant, 2013)—there is a need to develop additional methods to improve its treatment. The addition of *explicit case formulation* (CF) can help fine‐tune therapies and can be used effectively with manualized approaches such as CPT (Galovski et al., 2020). This method uses psychological theory to conceptualize an individual's presenting problem, thus facilitating the collaborative identification of treatment targets with clients and helping clinicians and clients address obstacles or barriers in therapy progress (Dudley et al., 2011). The use of CF is not new to the CPT approach and is included in past and current manuals (Resick et al., 2017; Resick, Monson, & Chard, 2024). However, research on the effectiveness of *explicit* CF, which provides clinicians with a framework to effectively plan and execute deviations from the CPT protocol when COTOs occur and help with decision‐making regarding deviations from the CPT protocol (e.g., to address a comorbid issue interfering with PTSD treatment), is still in its infancy. We use the term *explicit* to indicate that this is undertaken by actively involving the client in all aspects of these processes. Education on the use of formulation in standard CPT, labelled *cognitive case conceptualization*, is included in the latest CPT manual (Resick, Monson, & Chard, 2024). However, the advice in the manual and accompanying conceptualization form are clearly intended to help clinicians, not clients, in personalizing CPT; CF is not implemented directly or actively with clients.

In one study, Nixon and Bralo (2019) integrated explicit CF into CPT (CPT‐CF) in a mixed‐trauma PTSD sample. The authors found that CPT‐CF led to significant reductions in PTSD and depressive symptom severity, with sizeable effects (*d*s = 1.10–1.92), and improvements were maintained at a 3‐month follow‐up. Additionally, most participants achieved good end‐state functioning for PTSD (i.e., clinically reliable change, falling below clinical cutoffs) by posttreatment and follow‐up (62%–69%), indicating that the addition of CF did not derail therapy or hinder the positive outcomes of CPT. Notably, the disengagement rate was 17%, markedly lower than the 34% rate observed with standard CPT noted earlier. Clients reported good therapeutic alliance and acceptability of the CF approach. The authors argued that the approach effectively addresses both clinician‐level barriers (e.g., fears of inflexibility of manualized treatments) and client‐level barriers (e.g., comorbidities, complex clinical presentations) to the implementation of effective evidence‐based therapies.

Although promising, the study by Nixon and Bralo (2019) requires replication, and it is unclear whether this approach is as effective with first responders and veterans. As outlined, these are populations with higher rates of PTSD than the general population and may present specific challenges to treatment (e.g., stigma and engagement, comorbidities including anger, substance misuse); additionally, these populations have shown attenuated response to PTSD treatment. Accordingly, we aimed to extend on the work of Nixon and Bralo (2019), testing the efficacy of CPT‐CF in an open trial with first responders and veterans. In line with prior CPT‐CF work, we expected to see clinically significant reductions of PTSD and depressive symptoms, changes in other symptoms (e.g., problematic alcohol use), high therapy retention, and comparable levels of satisfaction with CF and the therapeutic process (i.e., therapeutic alliance).

## METHOD

### Participants

Participants were first responders (e.g., police, paramedics, and fire personnel, in both paid and unpaid professions; *n* = 27) or veterans (*n* = 2) who self‐referred or were referred by various services and professionals. The study was advertised through information sent to relevant local agencies and referrers, websites (study‐specific site, clinical trial registration site), and word of mouth. To be included, participants had to meet the full criteria for a PTSD diagnosis per the *Diagnostic and Statistical Manual of Mental Disorders* (5th ed., text rev.; *DSM*‐*5‐TR*; American Psychiatric Association, 2022). Exclusion criteria were (a) inadequate comprehension of English, (b) moderate‐to‐severe traumatic brain injury, (c) uncontrolled psychosis or substance dependence, (d) engaging in concurrent active trauma‐focused therapy, and (e) significant risk of harm (e.g., current domestic violence situation, active suicidality). In total, 60 participants contacted the research team. Approximately half of these individuals (*n* = 31) were not accepted or were excluded after the initial assessment (uncontactable/withdrew interest, *n* = 11; already in active therapy or similar study, *n* = 7; did not meet *DSM‐5* PTSD Criterion A or had low symptom levels, *n* = 13), with 29 being accepted into the study. A total of 23 participants completed treatment, one did not start therapy (moved out of state), and five disengaged during treatment or were considered dropouts (one due to a competing therapy program, two due to reported anxiety from sessions, two for unknown reasons). The sample included 22 men and seven women, and the mean participant age was 47.66 years (*SD* = 10.55, range: 27–70 years). Participants largely identified as White (*n* = 27, minority: *n* = 2) and had completed an average of 13.55 years of education (*SD* = 1.99). All participants met the criteria for PTSD, and a large number had other comorbidities: mood disorder (*n* = 17), substance use disorder (*n =* 12), social anxiety disorder (*n =* 10), obsessive–compulsive disorder (*n =* 1), panic disorder (*n =* 2), agoraphobia (*n =* 2), somatic symptom disorder (*n =* 3), and binge‐eating disorder (*n =* 1). The primary index trauma types were fatal transport or accident events (*n =* 13), fatal interpersonal events (*n =* 9), assault (*n =* 3), sexual assault (*n =* 3), and bushfires (*n =* 1). The average time since the reported index or first traumatic event was 11.72 years (*SD* = 11.31, range: 1 month–39 years), with approximately 48% of reported index or first traumatic events having occurred more than 10 years previously. All participants had experienced or been exposed to multiple traumatic events, with 21%–29% reporting 20 or more occasions of exposure to sudden or violent death. The final sample was sufficiently powered to detect a medium treatment effect size (i.e., G*Power [Faul et al., 2009]; power = 80%, one‐tailed related samples *t*‐test).

### Procedure

#### Overview

Participants underwent screening and, following informed consent, completed a pretreatment assessment that included diagnostic interviews and self‐report measures, after which they commenced therapy (in‐person). Posttreatment assessments were conducted 2 weeks after the conclusion of therapy and again at 3 months posttreatment, with interviewers unaware of the participant's treatment status (i.e., progress during therapy, treatment completion vs. dropout). Participants did not receive reimbursement for completing study assessments. This study followed an open trial design with assessments at pretreatment, posttreatment, and 3‐month follow‐up. Ethics was granted by the Southern Adelaide Clinical Human Research Ethics Committee (reference number: HREC/14/SAC/270), and the study was preregistered (Australian and New Zealand Clinical Trials Registry, ACTRN12619000503123).

#### Therapy

Participants were offered up to 25 weekly sessions of CPT (Resick et al., 2017; Resick, Monson, & Chard, 2024) with CF (*M* = 11.52, *Mdn* = 12, range: 0–22). CPT is a trauma‐focused therapy with a key focus on identifying and challenging unhelpful beliefs (labelled *stuck points*) that began after or were strengthened by the index traumatic event. Key elements of therapy include psychoeducation, a written statement detailing how the event has impacted the client's life, the identification of stuck points, an introduction to the cognitive model, challenging thoughts (stuck points), and an exploration of five trauma‐related themes (i.e., safety, trust, power/control, esteem, intimacy) that commonly affect individuals with PTSD. Clinicians use Socratic dialogue and various worksheets throughout therapy. The use of a written trauma account is optional and was used with four clients in the current sample.

The CF component was based on that used in Nixon and Bralo (2019, online supplement), with more detailed descriptions of this approach found in Galovski et al. (2025, 2020). CPT was delivered according to the manual, with the following CF‐related modifications. Session 1 was 60–90 min, during which the therapist and client collaboratively created a CF diagram detailing the development and maintenance of PTSD specific to the individual. This included identifying proximal and distal factors that might influence the client's current adjustment as well as their strengths and goals. Information gathered during the assessment and first session was summarized in a therapeutic letter that was given to the client at the beginning of Session 2. When issues arose that hindered treatment progress or when there was a lack of treatment response as indicated by weekly monitoring, CF was used to plan modifications and/or deviations from the CPT protocol. Evidence‐based cognitive behavioral techniques (or those consistent with this approach) were utilized to address issues (e.g., insomnia, depression, motivational interviewing). The client and therapist collaboratively determined whether deviation from the protocol was necessary, with the therapist also receiving input from the supervisor. Up to five sessions were permitted during each deviation; however, as seen previously (Nixon & Bralo, 2019), typically only one or two sessions were necessary. Deviations were coded as per Nixon and Bralo by an independent rater trained in CPT, with deviations or divergence considered as minor adjustments to CPT (e.g., psychoeducation regarding alcohol misuse), moderate adjustments (e.g., addressing interpersonal relationship problems for a portion of the session), or major adjustments (e.g., in‐depth use of non‐CPT techniques to address panic attacks). CPT completion was defined as completing all CPT content and a collaborative ending of therapy where there was mutual agreement that sufficient reductions in symptoms had been achieved.

#### Therapists and treatment fidelity

Therapy was delivered by five clinicians undergoing master's‐ or doctoral‐level postgraduate training. There was one exception to this: The first author, a registered clinical psychologist, provided therapy to one participant. Clinicians received weekly supervision by the first author, a CPT‐accredited trainer. Assessments and therapy sessions were recorded, and an independent CPT expert reviewed 21 sessions (across 10 clients) for adherence and competency (see Supplementary Materials). Therapy letters were also evaluated for four clients. Therapists delivered 100% of the essential components of CPT. Session competence and overall skill were rated on a scale of 1 (*poor*) to 7 (*excellent*), with mean therapist ratings of 5.33 (*SD* = 1.28) and 5.57 (*SD* = 1.17), respectively, thus falling between “good” and “very good” on the scale. The random tape selection included three tapes for which it was meaningful to assess therapist competency in CF. As with Nixon and Bralo (2019), this was done with a six‐item CF rating scale (Page et al., 2008), with possible ratings per item ranging from 1 (*deficient ability/inadequate*) to 5 (*high level of ability*). The mean score of 21.00 (*SD* = 7.00) from a total possible score of 30 reflected acceptable competency on this measure.

### Measures

The following well‐established measures were used at pretreatment, posttreatment, and at a 3‐month follow‐up, unless stated otherwise.

#### Diagnostic interviews

The Clinician‐Administered PTSD Scale for *DSM‐5* (CAPS‐5; Weathers, Blake, et al., 2013a), a structured interview, was used to diagnose PTSD according to *DSM‐5* criteria. The MINI International Neuropsychiatric Interview (MINI; Sheehan et al., 1997), which is aligned with *DSM*‐*IV* criteria, was used at pretreatment to determine the presence of comorbid conditions. Limited funding prevented interrater reliability checks; however, assessors were trained and monitored in the same fashion as in previous studies by the first author. This included completing online training (for the CAPS‐5) and the coding of unrelated tapes before clinical assessments. This has previously resulted in high interrater reliability (e.g., 92%–100% for PTSD diagnosis, 87%–100% for comorbid disorders; Angelakis et al., 2020; Nixon et al., 2016; Nixon & Bralo, 2019).

The CAPS‐5 (Weathers, Blake, et al., 2013a) is a 30‐item structured interview. The scale produces high interrater reliability (кs = .78–1.00) and test–retest reliability (к = .83) and has demonstrated convergent validity with *DSM‐IV* versions of the measure (*r* = .83) and the PTSD Checklist for *DSM‐5* (PCL‐5 [Weathers, Litz, et al., 2013]; *r* = .66; Weathers et al., 2018). Bryant et al. (2019) reported internal consistency produced by the CAPS‐5 in a sample of emergency service workers (Cronbach's α = .68). In the current sample, Cronbach's alpha was .83.

The MINI International Neuropsychiatric Interview (MINI; Sheehan et al., 1997) is a brief structured diagnostic interview for major Axis I psychiatric disorders and is based on *DSM‐IV* criteria. The interview produces good interrater reliability (кs > .75) and test–retest reliability (61%: к > .75) and has good concurrent validity with the Structured Clinical Interview for *DSM* Disorders—Patient Edition (SCID‐P) and the Composite International Diagnostic Interview (CIDI). Psychometric data specific to veterans or first responder samples have not been published; however, the MINI has been used extensively across clinical populations, and the interview has demonstrated reliability across diverse diagnostic categories.

#### Self‐report measures

##### Trauma history

A trauma history questionnaire adapted from Resick et al. (2002) was administered at pretreatment to assess the frequency and severity of past traumatic experiences. The measure comprised 12 potentially traumatic events (e.g., being physically assaulted) and asked participants to indicate the extent to which they experienced each in both childhood and adulthood (i.e., 24 questions in total). Participants were asked to rate each traumatic experience in terms of how often it occurred (0 = *never*, 6 = *more than 20 different times*) as well as how distressing they found the worst incident (1 = *minimally distressing*, 10 = *extremely distressing*). Items on the questionnaire are similar to other commonly used trauma history measures (e.g., the Life Events Checklist [LEC]; Weathers, Blake, et al., 2013b). Trauma history was measured in this fashion at this research site for the study to provide (a) consistency with information gathered in prior clinical trials and (b) relevant clinical information to the clinician. For example, most trauma history measures document the frequency of events but not the level of distress associated with each category of traumatic experience.

##### PTSD symptoms

The PTSD Checklist for *DSM‐5* (PCL‐5; Weathers, Litz, et al., 2013) is a 20‐item scale that indexes PTSD symptom severity over the past month as defined by the *DSM‐5*. Responses are rated on a 5‐point scale ranging from 0 (*not at all*) to 4 (*extremely*) and summed. A score of 31 or above is considered to reflect clinically significant PTSD symptom severity and a probable PTSD diagnosis (Blevins et al., 2015; Bovin et al., 2016). The total scale has produced good test–retest reliability (*r* = .82; Blevins et al., 2015) and internal consistency (Cronbach's αs = .94–.96) across both civilian (Blevins et al., 2015) and veteran and first responder samples (Bovin et al., 2016; McCall et al., 2023) and has demonstrated good convergent validity with the CAPS‐5 (*r* = .66; Weathers et al., 2018). In the current sample, Cronbach's alpha was .89.

##### Depressive symptoms

The Depression Anxiety and Stress Scale (DASS‐21; Lovibond & Lovibond, 1995) is a 21‐item self‐report questionnaire assessing symptoms of depression, anxiety, and stress. The total DASS‐21 scale produces excellent internal consistency (Depression subscale: Cronbach's α = .88, Anxiety subscale: Cronbach's α = .82, Stress subscale: Cronbach's α = .90), has demonstrated acceptable test–retest reliability (intraclass correlation coefficients [ICCs]: .75–.86; Kakemam et al., 2022), and has shown good convergent and discriminant validity (Henry & Crawford, 2005). Reffi et al. (2023) reported Cronbach's alphas of .90 (Depression subscale), .78 (Anxiety subscale), and .84 (Stress subscale) in a sample of first responders. Scores were doubled for the Depression subscale and used for analysis and reporting, which reflects the scoring of the full 42‐item DASS and use of the published clinical cutoffs for reliable change index (RCI) analyses (Table 2). In the current sample, Cronbach's alpha values were .91 for the Depression subscale, .84 for the Anxiety subscale, and .87 for the Stress subscale.

##### Quality of life

The Assessment of Quality of Life (AQoL‐8D; Richardson et al., 2014) is a 35‐item questionnaire with raw scores ranging from 35 to 176, where higher scores indicate better quality of life. The AQoL‐8D produces acceptable test–retest reliability (ICCs = .89–.91), demonstrates excellent internal consistency (Cronbach's αs > .89 across dimensions), and has excellent convergent validity with other measures of quality of life (*r*s = .68–.79). Data specific to first responder or veteran populations is unpublished at this time, but the total AQoL‐8D scale has shown good psychometric performance across various clinical samples and has been used extensively in Australian population research (e.g., Richardson et al., 2012). In the current sample, Cronbach's alpha was .93.

##### Negative posttrauma cognitions

The Posttraumatic Cognitions Inventory (PTCI; Foa et al., 1999) is a 36‐item self‐report questionnaire measuring trauma‐related thoughts and beliefs, with higher scores indicating more negative trauma‐related cognitions. The measure includes three subscales: Negative Cognitions about the Self (PTCI‐Self), Negative Cognitions About the World (PTCI‐World), and Self‐Blame (PTCI‐Blame). The subscales have demonstrated excellent internal consistency (Cronbach's αs = .86–.97) and good test–retest reliability (*r*s = .75–.89) and moderate‐to‐high correlations with PTSD symptom severity (PTCI‐Blame: *r* = .57, PTCI‐World: *r* = .69, PTCI‐Self: *r* = .78) and have discriminated between traumatized individuals with and without PTSD. Bryant et al. (2019) found internal consistency ranging from .81 to .90 across subscales in a sample of first responders. In the current sample, Cronbach's alpha was .96.

##### Sleep problems

The Insomnia Severity Index (ISI; Bastien et al., 2001) is a seven‐item self‐report measure assessing insomnia symptoms over the past 2 weeks. The total scale has demonstrated good internal consistency (Cronbach's αs = .74–.78) and adequate convergent validity with sleep diaries and polysomnographic measures (*r*s = .07–.45). Reffi et al. (2023) reported excellent internal consistency (Cronbach's α = .91) in a sample of first responders. In the current sample, Cronbach's alpha was .88.

##### Problematic alcohol use

The Alcohol Use Disorders Identification Test (AUDIT; Saunders et al., 1993) is a 12‐item self‐report questionnaire that assesses alcohol consumption, drinking behaviors, and alcohol‐related problems. The total scale has demonstrated good internal consistency (Cronbach's α = .82; Pradhan et al., 2012) and is significantly correlated with related constructs (e.g., Perceived Stress Scale [Cohen et al., 1983]; *r* = .16 [Lopez et al., 2019]). In samples of first responders, the AUDIT has produced sound internal consistency (Cronbach's αs = .79–.84; Bryant et al., 2019; Morrison et al., 2021; Reffi et al., 2023). In the current sample, Cronbach's alpha was .86.

##### Anger

The Dimensions of Anger Reactions (DAR‐5; Forbes et al., 2014) is a five‐item self‐report measure assessing anger intensity, frequency, and impact. The total scale shows excellent internal consistency (Cronbach's αs = .88–.90), acceptable test‐retest reliability (*r* = 73; Goulart et al., 2021), and strong convergent validity with other measures of anger (Forbes et al., 2014). In samples including first responders, DAR‐5 scores have demonstrated excellent internal consistency (Cronbach's αs = .86–.90; McCall et al., 2023; Miloslavich et al., 2023). Novaco et al. (2012) established the validity of the full DAR for combat veterans with multiple self‐rated measures of psychological distress, functional difficulties in multiple domains, and violence risk. In the current sample, Cronbach's alpha was .83.

##### Therapy process

Participants completed therapy‐process measures, including the Working Alliance Inventory–Short Form (WAI‐S; Tracey & Kokotovic, 1989), Credibility/Expectancy Questionnaire (CEQ; Devilly & Borkovec, 2000), and the University of Rhode Island Change Assessment–Trauma (URICA‐T; Hunt et al., 2006).

The WAI‐S was administered early in therapy (Session 2), in the middle of therapy (Session 6 or Session 10, due to flexible length of CPT), and at posttreatment. The measure includes 12 items assessing therapeutic alliance across three domains: task, bond, and goal agreement. The WAI‐S has demonstrated excellent internal consistency (Cronbach's α = .98) and test–retest reliability over a 2‐week period (*r* = .83; Tracey & Kokotovic). Kelly et al. (2022) reported good internal consistency (Cronbach's α = .81) in a sample of veterans. In the current sample, Cronbach's alpha was .94.

The CEQ was administered at Session 1 and posttreatment. The measure has demonstrated good internal consistency (Credibility: Cronbach's α = .86, Expectancy [standardized]: Cronbach's α = .90) and high test–retest reliability (*r* = .83) in a sample including veterans (Devilly & Borkovec, 2000). We used the scoring method described by Smeets et al. (2008), with total scores ranging from 6 to 54, with higher scores indicating more positive ratings. In the current sample, Cronbach's alpha was .87.

The URICA‐T (Hunt et al., 2006) is a modified version of the URICA for trauma populations. The total scale has demonstrated good internal consistency (Cronbach's α = .70) and positively correlates with treatment engagement (*r* = .32; Hunt et al., 2006). In a sample of veterans, overall reliability was adequate (Cronbach's α = .68), with Cronbach's alpha values for subscales ranging from .61 to .81 (Fleming et al., 2018). Cronbach's α = .87 (total score) in the current sample.

In addition, a five‐item Case Formulation Evaluation Questionnaire was adapted from that used by Nixon and Bralo (2019) and administered at Session 1 or 2 and posttreatment. Participants indicated their level of agreement on a scale of 1 (*totally disagree*) to 5 (*totally agree*) with statements that the formulation process was (a) understandable, (b) logical, (c) acceptable, (d) helpful, and (e) a good summary of their current difficulties. In the current sample, Cronbach's alpha was .89. More information is provided in the Supplementary Materials.

### Data analysis

Analyses were conducted on the intent‐to‐treat (ITT) sample (completer analyses are available upon request). Linear mixed‐model (LMM) analyses, using restricted maximum likelihood estimation for missing data, with planned comparisons (pre–post, pre–follow‐up) were conducted, and Hedges’ *g* effect sizes were calculated for within‐group changes. We calculated whether there was statistically significant individual participant change in PTSD and depressive symptom severity via the RCI method (Jacobson & Truax, 1991). RCI was calculated on ITT data, but due to challenges in estimating missing data for dichotomous variables, especially with modest sample sizes, analyses were conducted only on available data from the ITT sample. We defined remission, or good end‐state (GES), as reliable change in combination with the relevant symptom score falling below its cutoff (19 or lower for the CAPS‐5 or PCL‐5; 10 or lower for the DASS‐D), in line with prior research and recommendations (Matthews et al., 2022; Varker et al., 2020; Wachen et al., 2019). Further details for effect sizes and cutoff scores used can be found in the Supplementary Materials.

## RESULTS

Participants demonstrated significant symptom reductions over time, as summarized in Table 1 (ITT sample). Pairwise comparisons indicated that, generally, these significant pre–post and pre–follow‐up reductions were associated with large effects, PTSD: *g*s > 2.33, QoL: *g*s > 0.93, depressive symptoms: *g*s > 1.08. Modest, but still sizeable effects were observed for reductions in alcohol use, *g*s = 0.32–0.42, and improved sleep, *g*s = 0.71–0.82.

**TABLE 1:** Estimated means and standard errors for posttraumatic stress disorder (PTSD) symptoms, depressive symptom severity, and related outcomes across assessments

	Pretreatment		Posttreatment		Follow‐up		Pre–post		Pre–follow‐up		
Variable	*M*	*SE*	*M*	*SE*	*M*	*SE*	*g*	95% CI	*g*	95% CI	*F*(*df*)^b^
CAPS‐5	36.79	2.19	7.17	1.52	8.77	1.63	2.48	[1.43, 3.54]	2.35	[1.47, 3.22]	73.55 (2, 23.27)^***^
PCL‐5	44.69	2.51	10.49	2.05	12.70	2.21	2.50	[1.65, 2.24]	2.33	[1.57, 3.10]	87.44 (2, 25.08)^***^
DASS‐D	18.90	1.94	4.41	1.04	7.42	1.60	1.37	[0.63, 2.11]	1.08	[0.64, 1.53]	24.97 (2, 25.06)^***^
PTCI	141.14	7.63	95.04	8.84	89.90	7.67	1.11	[0.53, 1.68]	1.23	[0.67, 1.79]	16.16 (2, 24.21)^***^
ISI	14.07	1.13	9.70	1.24	9.03	1.72	0.71	[0.32, 1.09]	0.82	[0.41, 1.22]	11.86 (2, 24.22)^***^
AUDIT	9.79	1.29	7.52	1.22	6.90	1.14	0.32	[0.05, 0.60]	0.41	[0.06, 0.76]	4.52 (2, 23.06)^*^
DAR‐5	9.86	0.72	6.94	0.43	7.70	0.60	0.74	[0.27, 1.21]	0.55	[0.12, 0.98]	9.01 (2, 25.31)^**^
AQoL^a^	58.82	2.44	72.03	2.66	71.16	2.77	−0.99	[‐1.42, ‐0.56]	−0.93	[‐1.27, ‐0.59]	17.69 (2, 23.98)^***^
AQoL Utility^a^	0.47	0.03	0.67	0.04	0.65	0.04	−1.22	[‐1.72, ‐0.72]	−1.10	[‐1.47, ‐0.73]	16.53 (2, 24.10)^***^

*Note*: *N =* 29, intent‐to‐treat sample. CAPS‐5 = Clinician Administered PTSD Scale for *DSM‐5*; PCL‐5 = PTSD Checklist for *DSM‐5*; DASS‐D = Depression Anxiety Stress Scale–Depression subscale (scores doubled); PTCI = Posttraumatic Cognitions Inventory; ISI = Insomnia Severity Index; AUDIT = Alcohol Use Disorders Identification Test; DAR‐5 = Dimensions of Anger Reactions Questionnaire; AQoL = Assessment of Quality of Life; CI = confidence interval; *df* = degrees of freedom.

^a^ An increase in score indicates an improvement in quality of life or capabilities.

^b^ *F* = main effect of time.

* *p* < .05. **p < .01. ***p < .001.

We saw that most participants no longer met the criteria for PTSD at posttreatment and follow‐up and demonstrated reliable (i.e., statistical) individual change through RCI analyses (Table 2). These changes were most robust for PTSD outcomes over time, with a smaller proportion of the group maintaining reliable change and remission for depressive symptoms, although given the modest sample size, an absolute difference of two participants between categories can somewhat artificially inflate data when expressed as proportions.

**TABLE 2:** Loss of posttraumatic stress disorder diagnosis, reliable change (response to treatment), and good end‐state functioning status (remission) in the intent‐to‐treat sample

Variable	Posttreatment			Follow‐up		
	Total			Total		
Outcome	*n*	*n*	%	*n*	*n*	%
No PTSD (CAPS diagnosis)	23	22	95.7	24	24	100.0
Treatment response^a^, ^b^						
PTSD (CAPS)	23	20	87.0	22	19	86.4
PTSD (PCL)	24	23	95.8	24	21	87.0
Depression (DASS‐D)	12	10	83.3	11	6	54.5
Good end‐state functioning^a^, ^c^						
PTSD (CAPS)	23	20	87.0	23	19	82.6
PTSD (PCL)	23	22	95.7	22	20	90.1
Depression (DASS‐D)	12	8	66.6	11	5	45.5

*Note*: PTSD = posttraumatic stress disorder; CAPS‐5 = Clinician Administered PTSD Scale for *DSM‐5*; PCL‐5 = PTSD Checklist for *DSM‐5*; DASS‐D = Depression Anxiety Stress Scale–Depression subscale.

^a^ Only participants with scores in the clinical range at pretreatment were included in the analysis.

^b^ Reliable (i.e., statistical) change.

^c^ Reliable change *and* symptom severity score below good end‐state cut‐off (≤ 19 for CAPS or PCL‐5; < 10 for DASS‐D).

Across the sample, there were three cases of moderate deviations from CPT and four major deviations. In the former, this reflected the use of behavioral experiments to further test beliefs and stuck points, more substantial use of behavioral activation for depression (beyond that normally undertaken in CPT), and/or strategies to manage problematic drinking. Major deviations were characterized by the systematic use of in vivo exposure (addressing trauma‐related avoidance), sessions to address motivation or engagement at a therapy impasse, and methods to address self‐harm and suicidality. Overall, these seven participants demonstrated good outcomes at posttreatment that were maintained at 3‐month follow‐up. This included GES (remission) for PTSD and/or depressive symptoms (*n* = 3) or clinically meaningful responses (*n* = 2). One participant did not maintain their treatment response at follow‐up, and the other disengaged and was not available for follow‐up. We examined whether baseline symptom scores, number of comorbid disorders, readiness to change, or treatment credibility ratings might present useful indicators of characteristics associated with later deviations during CPT. However, only minimal nonsignificant differences were observed between participants with moderate or major deviations and those with minor deviations or no deviations, *p*s = .277–.934. Unsurprisingly, individuals with moderate or major deviations received more sessions than other participants (*M* = 16.57 vs. *M* = 9.91), *p* = .003 (see Supplementary Materials).

Regarding therapy process measures, we observed a significant increase in working alliance during therapy, with therapy credibility and usefulness of the CF approach ratings remaining stable (Table 3). The increase in working alliance from early in therapy to the end of therapy reflected a large effect, *g* = 0.81, and the medium‐sized increase in CF, *g* = 0.42, while not statistically significant, might have reflected power or sample‐size issues.

**TABLE 3:** Estimated means and standard errors for working alliance, treatment credibility, and evaluation of case formulation across assessments

		Baseline		Mid‐treatment		Posttreatment		Baseline^a^–posttreatment		
Measure	*n*	*M*	*SE*	*M*	*SE*	*M*	*SE*	*g*	95% CI	*F*(*df*)^c^
WAI‐S	25	65.99	1.59	68.75	1.33	72.51	1.36	0.81	[0.41, 1.21]	16.45 (2, 21.50)^***^
Credible^b^	26	43.58	1.61	_		45.57	1.67	0.24	[‐0.21, 0.69]	1.14 (1, 20.91)
CF‐Eval^b^	26	20.70	0.45	_		21.67	0.63	0.42	[‐0.16, 1.00]	3.22 (1, 22.27)

*Note*. WAI‐S = Working Alliance Inventory–Short Form; Credible = Treatment Credibility/Expectancy Questionnaire; CF‐Eval = Case Formulation Evaluation. Higher scores reflect more favorable reports on all measures.

^a^ Baseline: Treatment Session 1 for credibility and Session 2 for working alliance and case formulation evaluation.

^b^ Only assessed at baseline and posttreatment.

^c^ *F* = main effect of time.

*** *p* < .001.

## DISCUSSION

Our findings examining the efficacy and acceptability of CPT with explicit CF in a largely first responder sample replicated earlier findings of this approach in a civilian trauma group (Nixon & Bralo, 2019). We observed large reductions in PTSD symptom severity (*g*s > 2.33) and depressive symptom severity (*g*s > 1.08). Improvements in quality of life were substantial (*g*s > 0.93) and modest, but still‐sizeable effects were observed for reductions in alcohol use (*g*s = 0.32–0.42) and improved sleep (*g*s = 0.71–0.82). We also saw reasonable therapy retention (approximately 82% of treatment starters), strong therapeutic alliance, and client satisfaction with the CF process that was comparable to that seen in Nixon and Bralo (2019). Diagnostic and response to treatment outcomes for PTSD were very positive, with more than 95% of participants no longer meeting the criteria for PTSD at posttreatment and 3‐month follow‐up. Although CPT is an established therapy with a significant evidence base (Resick, LoSavio, et al., 2024), our findings are noteworthy given the mostly male first responder sample, with more than 20% of the sample reporting 20 or more exposures to sudden or violent death in the course of their employment. A motivation for our study was the known attenuated response to PTSD treatments, including CPT, for men and veterans. Our findings lend further support to the possibility of improved outcomes in groups such as these by being able to flexibly target challenges to optimal outcomes through deviations from the protocol guided by judicious clinical decisions (i.e., CF). This work adds to the accumulating evidence and resources available to support clinicians to effectively conduct therapy with complex clinical presentations as seen in PTSD (see Galovski et al., 2020; Galovski, Nixon, & Kehle‐Forbes, 2024; Goodnight et al., 2019) and adds to what is a still a modest literature with respect to interventions tested specifically within first responder samples (Bahji et al., 2022).

Our sample was predominantly made up of first responders, with their outcomes comparing favourably with those in prior studies addressing PTSD in the same group, with larger effects than those reported in a recent review (Bahji et al., 2022). The observed outcomes are also generally in line with randomized trials using exposure‐based cognitive behavioral therapy (CBT) among Australian first responders with levels of clinical severity similar to participants in our sample (Bryant et al., 2025, 2019), although rates of PTSD at posttreatment were somewhat higher in those studies (i.e., ∼32% [Bryant et al., 2019]; 13.6%–23.2% [Bryant et al., 2025]) compared with our findings (less than 5%). Using a lower‐intensity CBT approach delivered online to a large sample of Canadian first responders, McCall et al. (2023) reported significant improvements in PTSD and depression (effect sizes > 0.70). These reflect relatively substantial effects despite the lower dose of intervention and therapist input. Relatedly, although the primary goal of CPT is to treat PTSD, it is worth highlighting that we saw substantial improvements on secondary measures and quality of life that were not universally targeted in treatment. This is important given calls for PTSD trials to report on and address outcomes beyond PTSD (Varker et al., 2020). We did note that in our sample, only approximately half of the participants were above the clinical cutoff for depressive symptoms, although most showed clinically reliable reductions immediately following treatment. The small sample precludes firm conclusions as to whether depressive symptoms were more likely to return at follow‐up; however, in some cases, targeting depression more directly during or immediately after CPT might be beneficial (see Angelakis et al., 2020; Nixon & Elizabeth, 2025; Resick et al., 2021).

Our uncontrolled design precludes firm conclusions regarding what was responsible for the magnitude of our effects; however, it is likely that the flexible approach, including the number of sessions, which allowed time for deviations if required, was one contributor to successful outcomes. Prior CPT studies that have had flexibility in the length of treatment have similarly shown positive outcomes relative to those seen in fixed 12‐session CPT trials (e.g., Galovski et al., 2012; Resick et al., 2021). It is noteworthy that a generally good treatment response was seen among participants for whom deviations were made, suggesting that when undertaken judiciously, these divergences did not derail treatment (e.g., by colluding with a participant's avoidance of trauma‐related material). In a similar vein, it is also noteworthy that standard CPT (without deviations) was beneficial for most participants. This does not mean some clinical challenges were not present in these presentations but does speak to the utility of standard CPT to address these challenges as part of the normal protocol. Our observation that few baseline variables predicted whether divergences were required underscores the challenges clinicians face in decision‐making when nonresponse is observed. As documented by Nixon et al. (2021), it is hard to predict early—or even after eight sessions—who is ultimately on a trajectory for positive outcomes when using variables easily accessible to clinicians (e.g., clinical severity, trauma types and history). Accordingly, further research is needed to enable the earlier detection of factors associated with poor response and how CF can be best used to ensure optimal treatment outcomes.

We acknowledge several study limitations not already mentioned. The sample size was modest, which precluded an extensive examination of outcome predictors, including whether the impact of CF varied depending on the clinical issue being addressed. Participants were generally White men, indicating the need for further investigation in more heterogeneous samples and in light of known barriers to treatment for underserved and/or minority groups. The 3‐month follow‐up period was relatively brief; thus, it is unknown whether positive clinical outcomes were maintained, although CPT studies with follow‐ups of 6–9 months or even 5–10 years have shown that posttreatment gains generally remain (see Resick et al., 2012).

In conclusion, and with the above caveats in mind, our study has replicated earlier work (e.g., Bralo & Nixon, 2019) demonstrating the potential usefulness of explicit CF within CPT for first responders. This was associated with good retention, positive clinical outcomes, and improved quality of life, and was seen by participants as a credible and acceptable component of therapy. This and earlier work have laid the foundation for ongoing investigations of CF in CPT, including a large, randomized trial with veterans (Galovski McSweeney, et al., 2024), with the hope that evidence‐based interventions such as CPT can be further enhanced to increase their effectiveness and applicability to more trauma populations.

## AUTHOR NOTE

This work was supported by a Prabha Sheshadri Grant awarded by The Road Home and The Hospital Research Foundation.

Reginald D.V. Nixon occasionally conducts clinical workshops for treating posttraumatic stress disorder (PTSD), including cognitive processing therapy (CPT), and receives royalties from a book on CPT. Tara E. Galovski occasionally conducts clinical workshops for treating PTSD, including CPT, and only reports conflicts of interest for intellectual property of the book on CPT. Neither of these authors received consultancies as part of this research. David Forbes has no conflicts of interest to declare.

## OPEN PRACTICES STATEMENT

The preregistration for this study can be accessed at https://www.anzctr.org.au/ (Trial No. ACTRN12619000503123). The data have not been made available on a permanent third‐party archive; requests for the data or materials can be sent via email to the lead author at reg.nixon@flinders.edu.au.

## Supporting information

SUPPORTING INFORMATION
