# Supplementary material for: Cognitive processing therapy for posttraumatic stress disorder in first responders and veterans: Flexing the approach with explicit case formulation
Source: J Trauma Stress. 2025 Sep 14;38(6):1045–58. doi: 10.1002/jts.70005 (PMC12711452; doi:10.1002/jts.70005)
Supplement: Supplementary file 1 — SUPPORTING INFORMATION [file JTS-38-1045-s001.docx]

**Supplement to**: Reginald D.V. Nixon, David Forbes, & Tara E. Galovski. (2025). Cognitive Processing Therapy for posttraumatic stress disorder in first responders and veterans: Flexing the approach with explicit case formulation. *Journal of Traumatic Stress*, doi: <https://doi.org/10.1002/jts.70005>

**Online Supplemental Material**

**Treatment Fidelity and Case Formulation Assessment**

The forms and procedures from Nixon and Bralo (2019) were used for this study. Specifically, the independent CPT expert used the Cognitive Processing Therapy (CPT) Therapist Adherence and Competence Protocol. This protocol outlines the components of CPT, session by session, for which each is rated as present or not (adherence) and for which the degree of competence demonstrated is similarly scored on a on a 7-point scale (1 = *poor*, 7 = *excellent*). The protocol was based on iterations of previously CPT fidelity checking protocols used in randomized CPT trials (e.g., Forbes et al., 2012; Monson et al., 2006). Therapists are also rated for essential elements of therapy that are not unique to CPT (e.g., genuineness, warmth, accurate empathy, agenda setting, homework review etc.). An overall rating of therapist skill for the rated session is also made on the 1-7 point scale.

The Case Formulation Rating Scale (Page et al., 2008) is a 6-item measure that assesses the degree to which the clinician addresses core elements of case formulation, including identifying presenting problems, predisposing, precipitating and perpetuating factors, putting together a provisional conceptualisation and identifying treatment interfering issues or problems as well as client strengths. The CPT expert made ratings on each item, where the scale ranged between 1 to 5. Scores of 1 reflect deficient ability/inadequate skill, and scores of 5 indicate high level of ability. For example, on the problem list item, a score of 5 reflects that all relevant problems have been identified, and that there is clear distinction between primary and secondary issues, whereas a score of 1 would reflect that some relevant problems might be identified but irrelevant factors are noted, and a primary problem has been missed. The measure is summed with scores ranging from 1 – 30, with higher scores reflecting higher skills / better formulation.

**Data Analysis**

When calculating effect sizes (Hedges’ *g*), baseline *SD* (rather than a pooled *SD*) was used in calculating unbiased estimate of effects as it provides a better estimate of the population variances since it has not been affected by the intervention (Goulet-Pelletier & Cousineau, 2018). The PCL cut-off used to indicate remission (PCL-5 ≤ 19) was lower than a recently reported clinical cut-off of 41 for first responders (Morrison et al., 2021) however the latter reflects probable versus non-probable PTSD; the score used in the present data reflects probable remission. RCI was calculated on ITT data but due to challenges in estimating missing data for dichotomous variables, especially with modest sample sizes, analysis was conducted only on available data from the ITT sample.

**Characteristics associated with later deviations during CPT**

As documented in Table S1, there were minimal differences in baseline clinical characteristics, readiness to change, or treatment credibility ratings between those with moderate/major deviations and those with minor/none.

Table S1

*Clinical and therapy characteristic differences between participants as a function of level of CPT deviation.*

|  | None / minor deviations (*n* = 7) | |  | Moderate / major deviations (*n* = 22) | |  |  |
| --- | --- | --- | --- | --- | --- | --- | --- |
| Measure | *M* | *SD* |  | *M* | *SD* |  | *F*(*df*) |
| CAPS-5 | 36.09 | 12.84 |  | 39.00 | 7.98 |  | 0.32 (1, 27) |
| PCL-5 | 44.18 | 13.66 |  | 46.29 | 14.08 |  | 0.12 (1, 27) |
| DASS-D | 19.36 | 10.87 |  | 17.43 | 9.64 |  | 0.18 (1, 27) |
| PTCI | 138.14 | 39.82 |  | 150.57 | 46.84 |  | 0.48 (1, 27) |
| ISI | 13.36 | 6.24 |  | 16.29 | 5.41 |  | 1.23 (1, 27) |
| AUDIT | 9.27 | 6.66 |  | 11.43 | 8.16 |  | 0.50 (1, 27) |
| DAR-5 | 10.18 | 4.12 |  | 8.86 | 3.13 |  | 0.61 (1, 27) |
| AQoL^a^ | 59.06 | 12.27 |  | 58.05 | 16.64 |  | 0.03 (1, 27) |
| AQoL Utility^a^ | 0.47 | 0.17 |  | 0.46 | 0.20 |  | 0.01 (1, 27) |
| No. Comorbid Dx | 1.73 | 1.86 |  | 2.57 | 1.90 |  | 1.10 (1, 27) |
| URICA-T | 2.34 | 0.71 |  | 2.52 | 0.74 |  | 0.32 (1, 27) |
| Time since trauma (years) | 11.33 | 11.87 |  | 12.94 | 10.05 |  | 0.10 (1, 27) |
| Credible | 43.79 | 8.45 |  | 42.16 | 6.26 |  | 0.16 (1, 22) |
| No. sessions | 9.91 | 4.70 |  | 16.57 | 4.32 |  | 11.06 (1, 27)** |

*Note.* CAPS-5 = Clinician Administered PTSD Scale for DSM-5; PCL-5 = PTSD Checklist for DSM-5; DASS-D = 21-item Depression Anxiety Stress Scale, Depression subscale; PTCI = Posttraumatic Cognitions Inventory; ISI = Insomnia Severity Index; AUDIT = Alcohol Use Disorders Identification Test; DAR-5 = Dimensions of Anger Reactions Questionnaire; AQoL = Assessment of Quality of Life; No. Comorbid Dx = number of non-PTSD diagnoses. Credible = Treatment Credibility/Expectancy Questionnaire; No. sessions = Number of CPT sessions received.

^a^ Higher scores indicate better quality of life or capabilities.

* *p* < .05; ** *p* < .01; *** *p* < .001.

**References not in main text**

Goulet-Pelletier, J. C., & Cousineau, D. (2018). A review of effect sizes and their confidence intervals, Part I: The Cohen’sd family. *The Quantitative Methods for Psychology*, *14*(4), 242–265. [https://doi.org/10.20982/tqmp.14.4.p242](https://psycnet.apa.org/doi/10.20982/tqmp.14.4.p242)
